# The liver is populated by a broad spectrum of markers for macrophages. In alcoholic hepatitis the macrophages are M1 and M2^☆^

**DOI:** 10.1016/j.yexmp.2013.09.004

**Published:** 2013-10-19

**Authors:** James Lee, B. French, T. Morgan, Samuel W. French

**Affiliations:** a Harbor-UCLA Medical Center Department of Pathology, Torrance, CA 90502, USA; b Veteran’s Administration Long Beach California, Department of Medicine, USA

**Keywords:** Alcoholic hepatitis, Macrophages, CD163, TLR-4

## Abstract

**Background:**

Liver cell injury in alcoholic hepatitis (AH) is in part, due to macrophage generated proinflammatory cytokines i.e., M1, M2a, M2b, and M2c might be involved in ALD. The T cell response to chemokines and cytokines differs not only when M1 and M2 macrophages are compared but even when individual M2 subtypes are profiled.

**Purpose:**

In AH, M1 monocytes in the blood show increased sensitivity in the TNF-α response to LPS. Immunohistochemistry (IHC) studies showed that the liver sinusoids in ALD are abundantly populated by CD163 expressing type 2 macrophages. In this report, we profile many of the molecules associated with M1 and M2 macrophages in livers with AH using IHC.

**Methods:**

Using immunofluorescent antibody-labeling, we profiled the receptors, cytokines and chemokines observed in M1, M2a, M2b, and M2c macrophages in liver biopsies from patients with AH.

**Results:**

The increased CD 163 expression found in previous studies was confirmed as well an additional macrophage phenotypic marker CD206, suggesting that AH pathogenesis at least partially involves M2a and M2c macrophages. TGF-β was found to be robustly over expressed by liver sinusoidal macrophages. Macrophage expression of the phenotypic markers TLR-2, TLR-4 and TLR-8 – found in both M1 and M2 macrophages – as well as the chemokines CCL-1 and CCL-18 was found. However, IRF-4, which is related to IL-4 production and M2a polarization as well as the cytokines CCL-1 and Il-1β and the chemokine CXCL-1 were also observed, suggesting that M2a and M2b also play a role in AH pathogenesis.

**Conclusion:**

Livers with AH show robust macrophage over expression of TGF-β, a growth factor more commonly associated with M2 type macrophages and mostly known for its fibrogenetic properties. However, our immunoprofiling of macrophage over expression also shows that AH is driven by receptors, interferons, and cytokines that are commonly associated not just with M2 macrophages, but with M1 as well. Thus, a complex interplay between different types of macrophages expressing a diverse array of molecules and receptors is involved in AH.

## Introduction

Liver cell injury in AH is in part due to macrophage generated proinflammatory cytokines and sinusoidal obstruction. The response of some phenotypic subtypes of macrophages (Kupffer cells) causes injury to hepatocytes by way of innate immune injury in response to endotoxin. This was found in rodent models of early alcoholic liver disease and possibly in AH in humans (Miller et al., 2011). These changes are increased in response to acute alcohol ingestion. They are responses that are reversible when ethanol ingestion is stopped in experimental alcohol fed rodent models. The question is: what macrophages are involved in chronic alcohol abuse in humans who have AH? Plasticity and functional polarization are hallmarks of different types of macrophages i.e., M1, M2a, M2b, and M2c which might be involved in AH.

This differential modulation of the type of macrophage–chemokine system integrates polarized macrophages in pathways of resistance to or promotion of immune-regulation, tissue repair and remodeling (Mellins et al., 2011). The T cell response to chemokines and cytokines differs when M1 and M2 macrophages are compared. M1 has a Th1 response to IFNα and LPS. M2a, b and c cause a Th2 response of immune-regulation, matrix deposition and remodeling. M2a is a response to IL-4 and 13, M2b is a response to TLR/IL-1R agonists, and M2c responds to 1L-10 and suppresses immune responses to tissue remodeling (Bleesing et al., 2007; Mellins et al., 2011). The type of macrophages located in the liver sinusoids determines the type of the inflammatory process in AH.

The question remains as to the type of macrophage response that exists in AH. Monocytes derived from blood have provided the basis of studies for LPS-sensitive cellular response to induce TNF-α expression (Hill et al., 1992) using isolated Kupffer cells and cell-cultured raw 264.7 cell-line (Gobejishvili et al., 2006). In this report, we used immunofluorescence antibodies against macrophage markers to more fully classify the type of macrophages involved in alcoholic hepatitis. Using immunofluorescent antibody-labeling, we profiled the proinflammatory markers and chemokines observed in M1, M2a, M2b, and M2c macrophages in liver biopsies from patients with AH.

## Methods

Eight archived liver biopsies diagnosed as alcoholic hepatitis and 2 archived control livers were used in order to study the type of molecules expressed by macrophages in liver sinusoids.

### Immunohistochemistry

Liver tissue was fixed in 10% buffered zinc formalin. These sections were either single or double stained using antibodies raised in rabbit, mouse, or goats (see Table 1 for list of antibodies and their origin). A Nikon 400 fluorescent microscope was used and morphometric monitoring was performed under three filters (i.e., FITC-green, Texas-red, and tricolor) using Nikon software. Photographs were taken of liver biopsies focusing on sinusoidal macrophages to determine the types of macrophages (i.e., M1, M2a, M2b, M2c) and the types of molecules that are expressed by these cells.

## Results and discussion

**Fig. 1:**
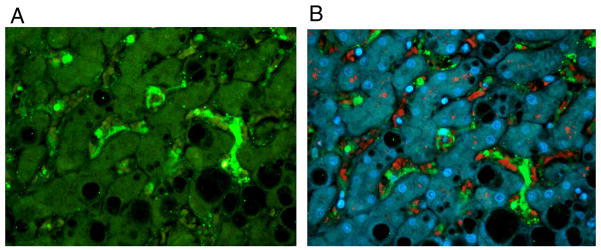
The antibody to F4/80 showed numerous positive macrophages in liver sinusoids staining green (A) in a patient with alcoholic hepatitis. The tricolor filter (B) showed that macrophages stain strongly positive alongside red blood cells (red colored). Magnification: A, B ×650. (For interpretation of the references to color in this figure legend, the reader is referred to the web version of this article.)

Using immunohistochemistry (IHC), we found robust F4/80 expression throughout the liver sinusoids (Fig. 1). F4/80 is a well characterized membrane protein expressed at high levels on the surface of various macrophages including Kupffer cells. In order to sub-classify these macrophages, immunostains for the most common chemokines, cytokines, receptor molecules and/or other phenotypic markers were performed. Table 2 lists the cytokines, chemokines, and phenotypic markers found to be over expressed using fluorescence antibody-labeling. Also listed (in red) are molecules often found to be over expressed in peripheral blood macrophages.

**Fig. 2:**
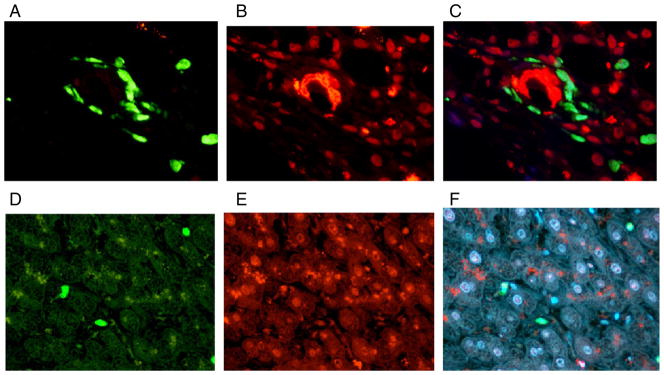
The antibody to CD163 showed numerous positive macrophages stained green (A) in a patient with severe alcoholic hepatitis and with Mallory–Denk body (MDB) formation. Stain for the antibody to ubiquitin stained the MDBs red (B) The tricolor filter showed that the CD163 positive macrophages surrounded the liver cell that formed the Mallory body (C) indicating that the macrophages are responding to the Mallory body affected liver cell. The control showed scattered CD163 positive macrophages (D–F). Magnification: A–C ×650. D–F ×433. (For interpretation of the references to color in this figure legend, the reader is referred to the web version of this article.)

**Fig. 3:**
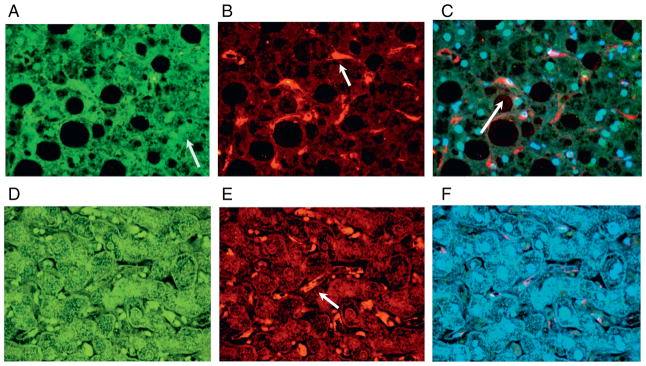
The antibody to CXCL9 showed numerous positive macrophages stained green (A, arrow) in a patient with alcoholic hepatitis. Stain for the antibodies to CD206 stained the macrophages red (B, arrow). Using the double stain with the tricolor filter showed that CD206 is positive in the macrophages (C, arrow). The control showed that CXCL-9 was not expressed by sinusoidal macrophages (D). The control showed sinusoidal CD206 positive macrophages (E). The control tricolor was negative (F). Magnification: A–F ×433. (For interpretation of the references to color in this figure legend, the reader is referred to the web version of this article.)

It was previously found in our lab that there was robust CD 163 expression observed in macrophages throughout the liver sinusoids (French et al). This result was confirmed using fluorescent antibodies (Fig. 2). In addition, CD206 expression by macrophages (Fig. 3) was also increased, which suggests that AH pathogenesis is driven partially by M2a and M2c macrophages.

In contrast to the spectrum of M1-derived chemokines, M2-derived chemokines promote recruitment of leukocytes involved in tissue repair and remodeling, allergy, resistance to helminth infection and tumor progression. However, livers with alcoholic hepatitis were mainly populated by M2 macrophages. This may not be surprising, as progression of liver fibrosis usually occurs due to repeated hepatic wound healing and regeneration, with a prominent feature being the recruitment of immunomodulatory cells including PMNs, monocytes, lymphocytes, and hepatic stellate cells (HSCs) to the site of liver injury. Hepatic resident macrophages and recruited inflammatory monocytes release factors including tumor necrosis factor α (TNF-α), platelet-derived growth factor (PDGF), reactive oxygen species (ROS), and transforming growth factor β (TGF-β) to activate HSCs. These components can drive the induction of liver fibrogenesis (Dooley and ten Dijke, 2012; Ghosh et al., 2013; Seki and Brenner, 2008).

Some bacterial pathogens have evolved sophisticated methods to prevent M1 polarization, neutralize microbicidal effectors of macrophages and promote M2 polarization. The persistence of bacterial pathogens in tissues and the chronic evolution of infectious diseases are linked to macrophage reprogramming toward heterogeneous M2 signatures (Benoit et al., 2008).

Based on numerous data from rat or mouse HSCs and animal models of liver damage, several conclusions about liver fibrosis can be made: (1) TGF-β is required for liver fibrosis and (2) the blunting of TGF-β signaling reduces fibrogenesis and is involved in HCC transformation (Chen et al., 2013; Dooley and ten Dijke, 2012; Meindl-Beinker et al., 2012). TGF-β has a pivotal role in orchestrating and regulating the corresponding phenotypes of chronic liver disease (Tsukamoto et al., 1990). Upon liver damage, TGF-β production is induced in non-parenchymal liver cells, e.g., granulocytes, macrophages (especially Kupffer cells) and hepatic stellate cells (Nakatsukasa et al., 1999), whereas, fully differentiated epithelial cells do not express TGF-β (Roth et al., 1998).

**Fig. 4:**
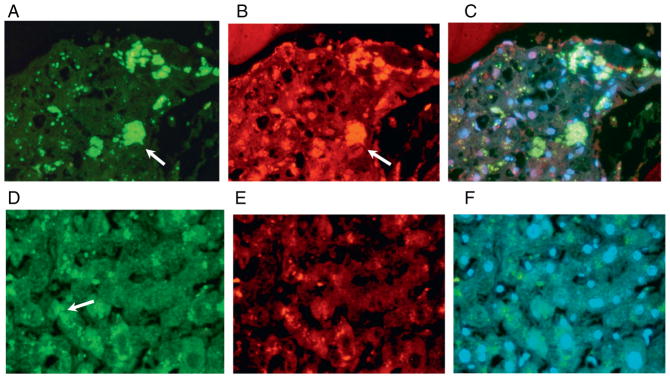
The antibodies to CD14 showed numerous positive macrophages stained green (A, arrow) in a patient with alcoholic hepatitis. The stain for the antibody to TGF-beta stained the macrophages red (B, arrow). Using the double stain with the tricolor filter showed that CD14 and TGF-beta co-localize in the macrophages (C, arrow). The control showed that CD14 (D) and TGF-beta (E) were not expressed by sinusoidal macrophages. The control tricolor was negative (F). In the controls, the only positive staining was for lipofuscin in hepatocytes (D, arrow). Magnification: A–F ×433. (For interpretation of the references to color in this figure legend, the reader is referred to the web version of this article.)

Thus, perhaps not so surprising then, was that TGF-β was found to be over expressed by liver macrophages in our immunofluorescence studies (Fig. 4B). TGF-β isoforms are multifunctional cytokines that play a central role in wound healing and in tissue repair. In general, the release and activation of TGF-β stimulate the production of various extracellular matrix proteins and inhibit the degradation of these matrix proteins. However, exceptions to these principles abound. These actions of TGF-β contribute to tissue repair, which under ideal circumstances leads to the restoration of normal tissue architecture and may involve a component of tissue fibrosis. In many diseases, excessive TGF-β contributes to a pathologic excess of tissue fibrosis that compromises normal organ function (Branton and Kopp, 1999).

In addition to fibrogenesis, TGF-β has also been shown to be able to arrest the cell cycle in G_1_ through induction of various cyclin inhibitors (Hannon and Beach, 1994; Polyak et al., 1994). More recently, our laboratory showed that livers with AH had increased expression of cell cycle inhibitors p21 and p27 and indeed had low proliferation index (French et al., 2012a), implicating a model whereby cell cycle cell arrest, impedance of liver regeneration, accumulation of DNA damage and subsequent oncogenetic effects as a mechanism for hepatocellular carcinoma transformation (French et al., 2012b). Our studies here showing such robust over expression of TGF-β by macrophages in AH thus provides an important link between liver macrophage accumulation and expression of inducers of cell cycle arrest and liver oncogenesis.

**Fig. 5:**
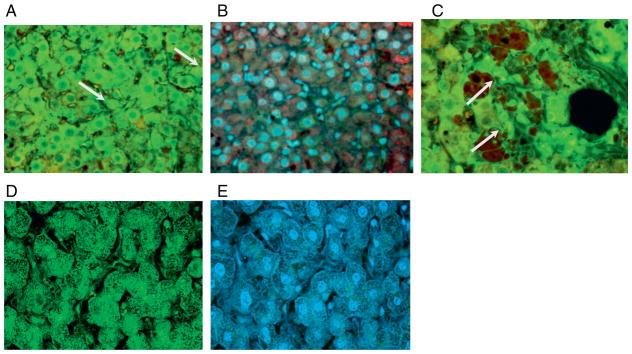
In livers with AH, both the macrophages (arrows) and the liver parenchymal cells show increased intensity of staining for TLR-4 indicating up regulation of expression (A and C). Tricolor filter (B and E). The control showed low intensity staining in liver cells (D). Magnification: A–C ×433, D and E ×650. (For interpretation of the references to color in this figure legend, the reader is referred to the web version of this article.)

**Fig. 6:**
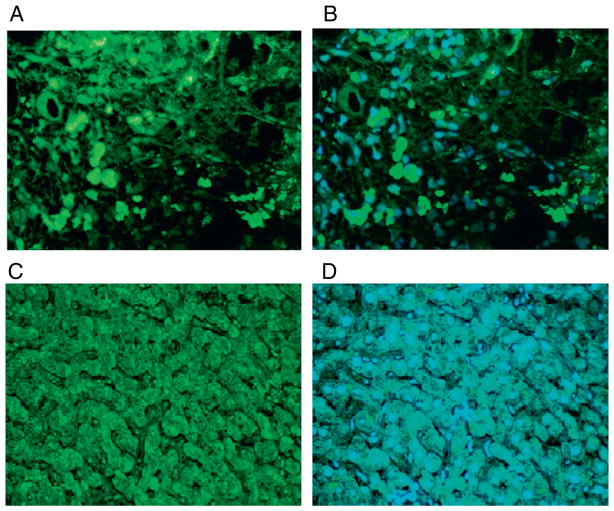
The antibody to TLR2 showed numerous positive macrophages stained green (A, arrow) in a patient with alcoholic hepatitis. Using the TLR2 stain with the tricolor filter showed that macrophages stained strongly positive (B, arrow). In the control, TLR-2 was not expressed by the sinusoidal macrophages (C). The control tricolor was also negative (D). Magnification: A–D ×217. (For interpretation of the references to color in this figure legend, the reader is referred to the web version of this article.)

**Fig. 7:**
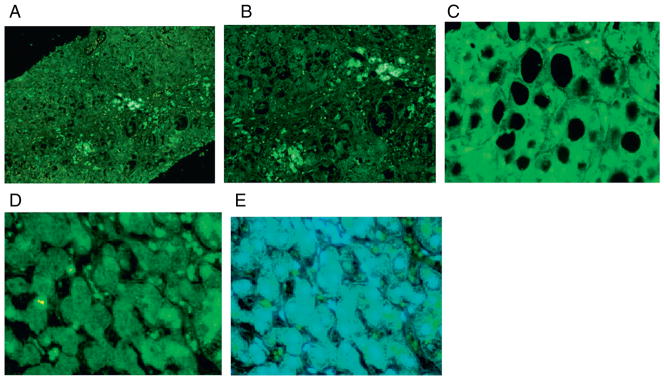
The antibody to TLR8 showed numerous positive macrophages stained green (A–C, arrows) in a patient with alcoholic hepatitis. Figure C shows numerous TLR-8 positive macrophages in an area of steatosis. The control showed that TLR-8 was not expressed by sinusoidal macrophages (D). The control tricolor was negative (E). Magnification: A ×108, B ×217, C ×650, D and E ×433. (For interpretation of the references to color in this figure legend, the reader is referred to the web version of this article.)

**Fig. 8:**
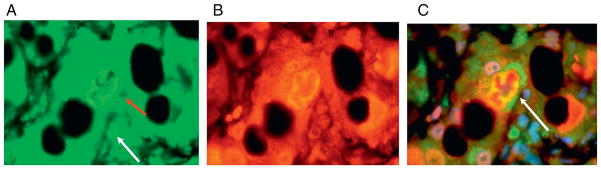
In a liver from a patient with alcoholic hepatitis, TLR4 was over expressed in the hepatocytes. Figure A shows that the macrophage (green arrow) and a hepatocyte (red arrow) are over expressing TLR4. Figure B shows ubiquitin staining the MDB red. Figure C shows co-localization of TLR4 with ubiquitin in the MDB-forming hepatocyte (yellow) which is over expressing TLR-4 (green rim around MDB, arrow). Magnification: A–C ×1083. (For interpretation of the references to color in this figure legend, the reader is referred to the web version of this article.)

Recognition of bacterial lipopolysaccharide (LPS) by the innate immune system elicits strong pro-inflammatory responses that can eventually cause a fatal sepsis syndrome in humans. This process is often associated with M1 macrophages and Toll-like receptors (TLRs). CD14 and TLR4 have been shown to form integral components of a membrane receptor complex that recognizes LPS (Dobrovolskaia and Vogel, 2002; Khachatoorian et al., 2013; Oliva et al., 2011; Triantafilou and Triantafilou, 2002). In mice, TLR4 over expression has been shown to induce tumor formation in hepatocellular carcinoma due to HCV (Chen et al., 2013). Furthermore, TLR2 and TLR4 expression is increased in hepatocytes, Kupffer cells, and peripheral monocytes of patients with chronic hepatitis C (Machida et al., 2012). Our studies indicate that CD14 (Fig. 4A) and TLR4 (Fig. 5) as well TLR2 (Fig. 6) and TLR8 (Fig. 7) are also all markedly up-regulated in AH, perhaps underscoring a common pathophysiology for various hepatic diseases. We also directly observe TLR4 over expression in hepatocytes that have formed Mallory–Denk bodies (MDBs) and thus, have disease progression (Fig. 8).

**Fig. 9:**
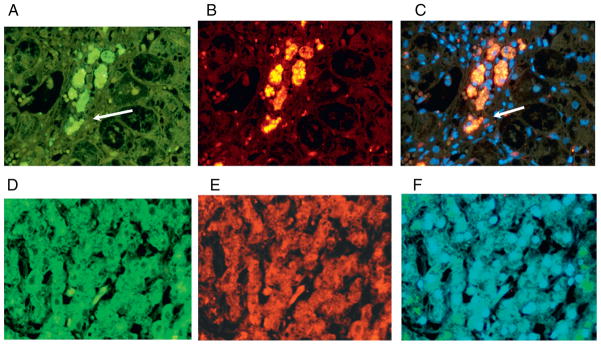
The antibody to IL-1β showed numerous positive macrophages stained green (A, arrow) in a patient with alcoholic hepatitis. Stain for the antibody to IRF4 stained the macrophages red (B). Using the double stain with the tricolor filter showed that IL-1 is co-localized with IRF4 (yellow) in the macrophages (C, arrow). The control showed sinusoidal Il-1 positive macrophages (D). The control showed that IRF4 was not expressed by sinusoidal macrophages (E). The control tricolor was negative (F). Magnification: A–F ×433. (For interpretation of the references to color in this figure legend, the reader is referred to the web version of this article.)

Our studies also show over expression of Il-1β (Fig. 9A), a well studied cytokine known for its pro-inflammatory properties. Clinically, Il-1β has been shown to be elevated in patients with alcoholic hepatitis (McClain et al., 1986). Furthermore, IL-1β induces MIP-1b expression in hepatic cells, perhaps underscoring a critical mechanism for continuous recruitment of inflammatory cells to liver and maintenance of inflammation (Zhang et al., 2003).

IRF-4 was also shown to be over expressed by liver sinusoidal macrophages (Fig. 9B) in AH. IRFs have been found to be key adaptor molecules involved in downstream processes initiated by TLRs in such processes as stimulation of proinflammatory cytokine and interferon production (Seki and Brenner, 2008). It remains to be seen what the role that IRF-4 plays in AH and why it was found in our studies to co-localize with Il-1 in the double IHC stain (Fig. 9C).

**Fig. 10:**
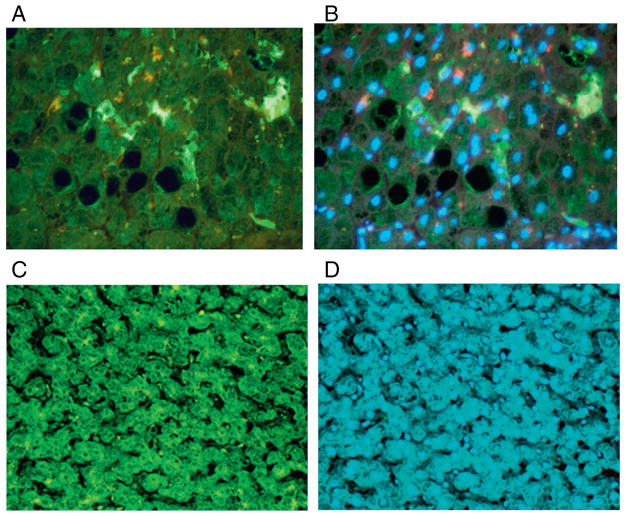
The antibody to CCL-1 showed numerous positive macrophages stained green (A) in a patient with alcoholic hepatitis. Using the TLR2 stain with the tricolor filter showed that macrophages stain strongly positive (B). The control showed that TLR-2 was not expressed by sinusoidal macrophages (C). The control tricolor was negative (D). Magnification: A–D ×433. (For interpretation of the references to color in this figure legend, the reader is referred to the web version of this article.)

Although the over expression of CD163 and CD206 would suggest that M2a or M2c macrophages are most involved in AH, our immunoprofiling indicates that CCL1 is also over expressed (Fig. 10). CCL1 was present in synovial fluids and macrophages in juvenile idiopathic arthritis (Mellins et al., 2011). Regulation of CCL1 in human monocytes is unique, with an obligate requirement of Fc gamma R engagement and co-stimulation by LPS and Il-1β, which were found to be over expressed by macrophages in our studies. CCL1 is a CC chemokine with a unique pattern of regulation associated with a distinct form of M2 (type 2, M2b) monocyte activation, which participates in M2b macrophages, providing a mechanism of amplification of Th2 polarization and immunoregulation.

## Conclusion

A complex network of varying macrophages expressing a diverse array of molecules is involved in liver pathogenesis in AH. Our studies here implicate several markers of acute inflammation. However, we also show that TGF-β, well-studied as a fibrogenetic factor as well an inducer of cell cycle arrest, also is over expressed in AH. It thus remains to be seen how acute inflammation, fibrosis, cell cycle arrest and DNA damage altogether lead to hepatocellular carcinoma transformation.

## Figures and Tables

**Table 1:** Antibodies used for immunoprofiling with animal source and company/vendor.

Antibody	Antibody type	Company/vendor
TGF β	Mouse	ISbio.com
IFNg	Mouse	Millipore
TNF-α	Rabbit	Millipore
Il-1β	Rabbit	Il-1B
Il-12A	Goat	ABCAM
Il-18	Rabbit	Life Span Biosciences Inc.
Il-4	Rabbit	ABCAM
Il-10	Rabbit	ABCAM
Il-6	Rabbit	ABCAM
CCL-8 (MCP2/ccl)	Rabbit	Biorbyt
CXCL 9	Rabbit	Abgent Inc. (San Diego)
IRF-5	Goat	ABCAM
CCL 13	Rabbit	Biorbyt
SR-A1	Rabbit	ABCAM
CCL-1	Rabbit	ABCAM
IRF-4	Rabbit	Cell Signalling
CXCL 1	Rabbit	Life Span Biosciences Inc.
CCL-18	Rabbit	Life Span Biosciences Inc.
S100-A8	Rabbit	Life Span Biosciences Inc.
Caspase 1	Rabbit	Epitomics
CD14	Mouse	Life Span Biosciences Inc.
TLR-2	Rabbit	Life Span Biosciences Inc.
TLR-4	Rabbit	Life Span Biosciences Inc.
TLR-8	Rabbit	Life Span Biosciences Inc.
F4/80	Rabbit	Proteintech Group
CD206	Rabbit	Life Span Biosciences Inc.

**Table 2:** Commonly found cytokines, chemokines, and phenotypic markers in various types of macrophages. In green are molecules found to be over expressed in AH using fluorescence antibody-labeling. In red are molecules often found to be over expressed in a specific macrophage subtype but were negative in our profiling of molecules involved in macrophage polarization for alcoholic hepatitis.

Subtype	Cytokines	Chemokines	Phenotypic markers	Additionally expressed molecules
M1	Negative: TNF-α, IFN-gamma	Negative: CXCL9	Negative:	Positive: TLR-2, TLR-4
	Positive: IL-1 β			
M2a	Negative: IL-10,	Negative: CCL13	Negative:	Negative:
		Positive: CCL18	Positive: CD163, CD206	
M2b	Negative: IL-10, TNFα	Negative:	Negative:	Negative:
	Positive: IL-1 β,	Positive: CCL1, CXCL1		
M2c	None known	Negative: CXCL9		
	Positive: TGF-β	Positive: CCL18	Positive: CD163, CD206	Positive: CD 14, TLR-8
